# Cholestatic Hepatitis with Small Duct Injury Associated with Celecoxib

**DOI:** 10.1155/2013/315479

**Published:** 2013-06-05

**Authors:** Suresh Kumar Nayudu, Shanti Badipatla, Masooma Niazi, Bhavna Balar

**Affiliations:** ^1^Division of Gastroenterology and Hepatology, Bronx Lebanon Hospital Center, Albert Einstein College of Medicine, Yeshiva University, Bronx, NY 10457, USA; ^2^Department of Medicine, Bronx Lebanon Hospital Center, Albert Einstein College of Medicine, Yeshiva University, Bronx, NY 10457, USA; ^3^Department of Pathology, Bronx Lebanon Hospital Center, Albert Einstein College of Medicine, Yeshiva University, Bronx, NY 10457, USA

## Abstract

Drug-induced liver injury (DILI) is a common clinical entity but is underreported due to various reasons. Cyclooxygenase-2 inhibitors like Celecoxib have been proven to be associated with lesser incidence of adverse drug reactions compared to other nonsteroidal anti-inflammatory drugs (NSAID). However, Celecoxib has been rarely reported to be associated with cholestasis and hepatitis. We present a young Hispanic female presented with cholestatic liver chemistries who has been taking Celecoxib for 3 weeks. Extensive workup did not support diagnosis of viral, autoimmune, or metabolic liver diseases. Liver biopsy revealed findings suggestive of secondary sclerosing cholangitis. Imaging studies were negative for large duct involvement, and endoscopy ruled out inflammatory bowel disease. Liver chemistries normalized after cessation of medication. We recommend that physician should be aware of this rare complication when prescribing Celecoxib.

## 1. Introduction 

Cyclooxygenase-2 (COX-2) inhibitors have been known to be associated with lower incidence of adverse events especially upper gastrointestinal complications compared to other nonsteroidal anti-inflammatory drugs (NSAID) [1]. Celecoxib, a known COX 2 inhibitor, has been reported to have beneficial effects against various malignancies in animal models [2–5]. Celecoxib has also been rarely associated with pancreatic and hepatic diseases especially acute and chronic cholestasis [6, 7]. Bile duct injury associated with drug-induced cholestasis has been reported very rarely with penicillin group of antibiotics [8, 9]. However, to the best of our knowledge, bile duct injury in association with Celecoxib has been reported only on one occasion in the literature [10]. We present a rare case of acute cholestatic hepatitis and small bile duct injury associated with Celecoxib in a young Hispanic woman. 

## 2. Case Presentation

A 34-year-old Hispanic woman presented to the emergency room (ER) with epigastric abdominal pain of 3 days duration. Pain is burning in nature, nonradiating and started insidiously 3 days prior to ER visit, which gradually worsened. She also reported nausea but denied vomiting, bowel, or urinary symptoms. She denied any fever, skin rashes, joint pains, loss of appetite, or loss of weight. She does not have any known medical conditions. She underwent breast cyst aspiration few years back. She never used tobacco, alcohol, or recreational drugs. She is sexually active with one partner using barrier contraceptive methods. She recently travelled to Dominican Republic and did not report any illness during her visit. However, she underwent a minor gynecological procedure for abnormal Papanicolaou (Pap) smear during her visit to Dominican Republic. She is not allergic to any medications.

On initial evaluation in ER, she was found to have jaundice and minimal epigastric tenderness. She received intravenous Ranitidine, and basic labs were drawn. She was found to have abnormal liver function tests and admitted to medical floor for further workup and management. Subsequently, medical team on floor requested gastroenterology evaluation.

On initial encounter with gastroenterology team on medical floor, the patient reported that her abdominal pain has resolved. She is well built and well-nourished woman, not in distress, and noted to have icterus. Her abdominal examination is benign without any tenderness, organomegaly or clinically detectable free fluid. Her cardiovascular, respiratory, and neurological examination is grossly normal. She does not have any skin rashes and scratch marks, and musculoskeletal examination was within normal limits.

Laboratory data revealed normocytic anemia with hemoglobin of 12, white cell count of 5.8, and platelets are within normal range. Her coagulation profile, electrolytes, blood urea nitrogen, and creatinine are normal. She had elevated alanine aminotransferase (ALT) of 458 units/liter, aspartate aminotransferase (AST) of 244 units/liter, alkaline phosphatase (ALP) of 231 units/liter, and total bilirubin of 3.4 milligrams/deciliter with predominant proportion of direct bilirubin, which is 2.8 milligrams/deciliter (Table 1). Her albumin and total protein are within normal limits. She is immune to hepatitis A and tested negative for hepatitis B and hepatitis C. Abdominal sonogram has not revealed any gallstones and has been reported as having common bile duct caliber of 3 mm. Computer tomogram (CT) of abdomen has not reported any abnormality. 

On further questioning, she revealed that she has been taking Celecoxib since three weeks as prescribed by her gynecologist in Dominican Republic. As there is no evidence of biliary obstruction or cholangitis, initial impression was probable drug-induced liver injury. However, further laboratory tests were ordered, including anemia workup and markers to diagnose common autoimmune and metabolic liver disorders. She has been advised to stop Celecoxib. She remained stable during her hospital stay and was discharged to home. She has been scheduled to follow up in gastroenterology clinic.

On followup in gastroenterology clinic, she remained asymptomatic. Her anemia workup revealed positive hemoglobin electrophoresis for sickle cell trait. Her ceruloplasmin levels are within normal limits, and transferrin saturation is 14% ruling out Wilson disease and hemochromatosis, respectively. She has been tested negative for anti-mitochondrial, anti-smooth muscle, and anti-liver kidney microsomal antibodies and was found to have normal immunoglobulin G levels. As workup has been negative for common viral, metabolic, and autoimmune liver diseases, patient was offered CT-guided liver biopsy. The patient underwent liver biopsy, which was uneventful. 

**Figure 1:**
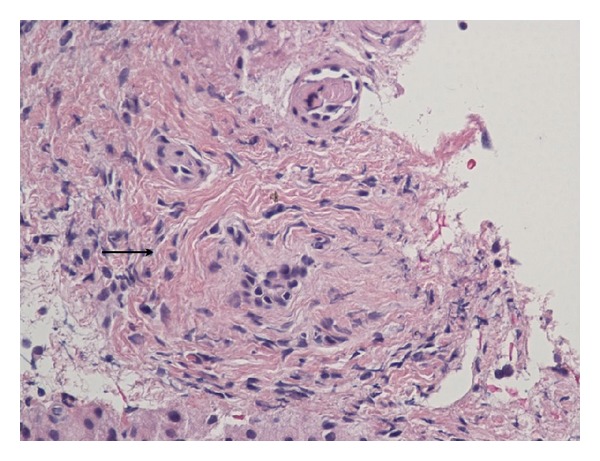
Liver biopsy portal tract and periductal fibrosis with narrowing of portal vein and it is branches.

Liver biopsy findings showed periductal fibrosis and findings suggestive of primary or secondary sclerosing cholangitis (Figure 1). On subsequent followup visits, liver function tests improved and reached baseline. However, in view of liver biopsy findings suggestive of sclerosing cholangitis, magnetic retrograde cholangiopancreatogram (MRCP) has been ordered which did not reveal any large bile duct abnormalities. The patient underwent esophagogastroduodenoscopy, which has not revealed any major lesions, and the biopsy tested negative for Helicobacter pylori. She underwent colonoscopy, which has not revealed any findings suggestive of inflammatory bowel disease. Currently, the patient is asymptomatic, and her liver function tests have been normal (Table 1).

## 3. Discussion

Nonsteroidal anti-inflammatory drugs (NSAIDs) have been noted to be associated with significant incidence of adverse drug reactions [1, 11]. However, COX-2 inhibitors like Celecoxib have been noted to be on the lower end of disease spectrum of adverse drug reactions especially upper gastrointestinal disorders [1]. It has also been noted that Celecoxib has been reported to have beneficial outcomes in gastrointestinal and liver neoplasms in animal models [12, 13]. 

However, Celecoxib has been rarely reported to be associated with acute and chronic cholestatic hepatitis [6, 7, 10, 14–16]. In very rare instances, abnormal liver chemistries persisted up to 18 months [6], and in one case, the clinical course worsened requiring liver transplantation [10].

Our case stressed the importance of obtaining detailed history of patient's medications and supplements. Cholestatic hepatitis has been reported in association with Celecoxib in the past; however, this case has rare feature of involving small biliary ducts and pathological findings resembling small duct sclerosing cholangitis [10]. Endoscopic workup has been carried out to rule out inflammatory bowel disease, which is a common association in patients with primary sclerosing cholangitis. Patient's liver chemistries normalized after cessation of medication, which supports the diagnosis of drug-induced liver injury (Table 1).

Drug-induced liver injury is common among general and hospitalized population, but due to various reasons, it has been underreported [11, 17]. However, abnormal liver chemistries always mandate complete workup to lead to particular diagnosis, including laboratory markers of viral, metabolic, and autoimmune liver diseases and ultimately liver biopsy guided by appropriate clinical situation [18, 19]. Detailed and appropriate history of medications and supplements always plays a major role in the work up of abnormal liver chemistries [18–20], which has been proved again in this case. Timely recognition and discontinuation of offending agent may prevent life-threatening complications [10] and improve patient outcomes. 

## Figures and Tables

**Table 1:** Liver chemistries.

	Day 1	Day 4	Day 16	1 month	6 months
Total protein^1^	7	7.3	8	7.6	7.7
Albumin^1^	4.3	4.1	4.7	4.3	4.5
Alanine aminotransferase^2^	458	402	147	10	12
Aspartate aminotransferase^2^	244	177	40	13	15
Alkaline phosphatase^2^	231	238	236	89	60
Total bilirubin^3^	3.4	3.4	1.1	0.7	0.4
Direct bilirubin^3^	2.8	2.2	0.4	0.3	0.1

^1^Grams/dL; ^2^milligrams/dL; ^3^units/liter.
